# Home-Based Digital Exercise Training Program to Improve Physical Function of Older Sepsis Survivors: Protocol of the HEAL Sepsis Randomized Clinical Trial

**DOI:** 10.2196/60270

**Published:** 2024-10-17

**Authors:** Rola S Zeidan, Margaret K Ohama, Natalia Evripidou, Stephen D Anton, Laith L Hamed, Yi Lin, Christiaan Leeuwenburgh, Faheem W Guirguis, Philip A Efron, Sheryl Flynn, Barbara Smith, Rhonda Bacher, Naveen Bakarasan, Juan Sarmiento Delgado, Robert T Mankowski

**Affiliations:** 1 Department of Physiology and Aging College of Medicine University of Florida Gainesville, FL United States; 2 Department of Health Outcomes and Biomedical Informatics College of Medicine University of Florida Gainesville, FL United States; 3 Department of Medicine Division of Gerontology, Geriatrics and Palliative Care University of Alabama at Birmingham Birmingham, AL United States; 4 Blue Marble Health Altadena, CA United States

**Keywords:** sepsis, physical function, exercise, rehabilitation, remotely delivered, aging

## Abstract

**Background:**

While sepsis, an exaggerated response to infection, can affect people of all age groups, it is more prevalent in middle-aged and older adults. Older adults suffer worse short-term and long-term outcomes than younger patients. Older sepsis survivors are commonly discharged to long-term acute care facilities, where they often die within 1 year. Those who return home from the hospital lose the momentum of physical function improvement after early inpatient rehabilitation, and often face exacerbation of comorbidities and decline in physical function. Additionally, patients who are discharged home often live at distant locations and are not able to commute to rehabilitation centers due to their poor health status. Therefore, remotely delivered exercise interventions tailored to this population hold promise to improve physical function safely and effectively after sepsis. However, this type of intervention has yet to be tested in this population.

**Objective:**

This study aims to assess the safety, feasibility, and ease of recruitment and retention of participants for a remotely delivered physical activity intervention for improving physical function in middle-aged and older sepsis survivors.

**Methods:**

The proposed intervention will be delivered through a digital health platform that comprises a patient-facing mobile app and a 12-week physical activity program specifically designed for middle-aged and older sepsis survivors with poor health status who may face challenges participating in traditional out-patient or community-based exercise interventions. This study is ongoing and plans to enroll 40 sepsis survivors aged 55 years and older who will be randomized to either a remotely delivered exercise intervention group or a control group (electronic health diary). Both groups will use a tablet containing the Health in Motion app (Blue Marble Health). The intervention group will receive a clinician-designed personalized avatar-guided home exercise program and reminders while the control group will self-report daily activities using the in-app health diary feature.

**Results:**

This study is the first to use a home-based, remotely monitored 12-week exercise program to improve physical function in sepsis survivors. This study will evaluate the safety, feasibility, and efficacy, providing the necessary knowledge to design and calculate power for future larger trials.

**Conclusions:**

This study will provide important information for planning a future randomized clinical trial to test the efficacy of a remotely delivered exercise intervention in this high-risk population.

**Trial Registration:**

ClinicalTrials.gov NCT05568511; https://clinicaltrials.gov/study/NCT05568511

**International Registered Report Identifier (IRRID):**

DERR1-10.2196/60270

## Introduction

Sepsis, an exaggerated response to an infection, affects all age groups [1]. However, adults aged 55 years and older are more commonly affected [2]. Despite reduced mortality, many sepsis survivors become critically ill with lasting low-grade multiple organ failure and systemic inflammation [3,4]. Older sepsis survivors are more likely to be discharged from the hospital to long-term acute care or skilled nursing facilities, and many require rehospitalization or die within 1 year [3]. Those who return home, often develop new or face exacerbation of preexisting comorbidities as well as a decline in physical function due to the lack of continuous physical function-enhancing interventions such as physical therapy (PT) or occupational therapy (OT) [1]. Postsepsis chronic systemic inflammation appears to perpetuate a decline in physical function in older adults by sustained muscle wasting leading to difficulties performing daily activities [1,4-6].

While early hospital-based PT and OT aim to improve physical function, patients often do not have access to structured interventions once discharged [7]. Thus, sedentary, home-bound sepsis survivors may be at high risk for stagnation or even worsening physical function, despite initial improvements in physical function gained during early rehabilitation. Therefore, there is a need for postdischarge exercise interventions customized to older sepsis survivors aimed at improving and sustaining their physical function at home. Aside from traditional home-based PT or OT available to a few individuals, a feasible home-based approach to improve the physical function of older sepsis survivors does not exist.

**Figure 1:**
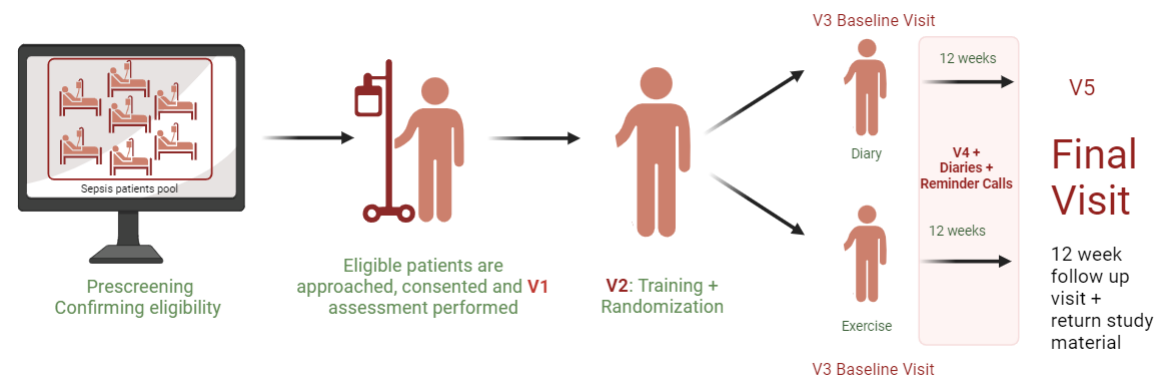
Conceptual model. V1: visit 1; V2: visit 2, V3: visit 3; V4; visit 4; V5: visit 5.

Current mobile health apps can deliver structured home-based exercise programs to reach daily activity goals. These mobile platforms can capture the most important outcomes, including physical function measures and adherence to exercise, entirely remotely via the mobile app. Data from assessments, exercise adherence, and response to exercise can be remotely monitored by the clinician at the clinical site who can remotely adjust the home exercise program to ensure the patient is performing the appropriate exercise dose and intensity. This unique approach can address the important issues of improving physical function in older sepsis survivors with poor health status, low physical function, and living in remote locations who are unlikely to participate in clinic-based exercise intervention programs. The goal of this paper is to describe a study protocol, that will test the feasibility and safety of a personalized 12-week digital, home-based, remotely monitored exercise intervention, compared to a control group who self-reports activities and does not receive exercise progression, in middle-aged and older sepsis survivors (Figure 1).

## Methods

### Study Design

To our knowledge, this will be the first single-blind, randomized clinical trial to evaluate the feasibility, safety, and efficacy of a 12-week remotely delivered intervention that administers avatar-guided exercise training with remote therapeutic monitoring to enhance physical function in middle-aged and older sepsis survivors. The study was registered with the database ClinicalTrials.gov (NCT05568511). The primary goals of this study are to test the safety and feasibility of this therapeutic intervention model. Safety is measured by the total number of adverse events occurring during the study period (primary) and the rate of adverse events per participant. The Health in Motion app (Blue Marble Health) allows for participants to report adverse experiences as well as illnesses, falls, and hospital visits. The participants will be encouraged to report all potential adverse events, and hospital records will be monitored regularly by study staff. Feasibility will be measured by adherence rates, completion rates, evaluation of reasons for withdrawal or noncompletion, and the number and nature of reports regarding technical difficulties that participants encounter while using the app. Efficacy will be measured by the mean change in the 30 second sit to stand test (30SSTS), the 4 stage balance test (4SBT), and timed up and go test (TUG) as measures of physical performance (secondary outcomes). The EQ-5D-5L will be used to capture self-reported health-related quality of life.

### Participants

This study will enroll 40 sepsis survivors aged 55 years and older with in-hospital short performance physical battery (SPPB) scores≤10 who will be discharged from the hospital and have access to Wi-Fi at home as the app requires it for proper use. Sepsis survivors who (1) will be discharged to a long-term facility, (2) will be involved in a structured rehabilitation program, (3) are unable to perform lower or upper body exercise, (4) have severe cardiac disease (New York Health Association class III or IV), (5) are significantly immunocompromised, (6) have been diagnosed with significant cognitive impairment, (7) have progressive degenerative neurological disease, (8) have severe pulmonary disease that requires steroid therapy or the use of supplemental oxygen, (9) are participating in another interventional study, or (10) are pregnant will be excluded. An overview of inclusion and exclusion criteria is provided in Textbox 1.

**Inclusion and exclusion criteria.**

**Inclusion criteria**

- Able to perform lower- and upper-body movements
- Sepsis survivor
- Aged 55 years and older
- Short performance physical battery (SPPB)≤10
- Being discharged to home from the hospital after surviving sepsis
- Willingness to be randomized to either treatment or control group
- Willingness to use the devices and technology in the study
- Willingness to participate in all study procedures

**Exclusion criteria**

- Failure to provide informed consent
- Pregnant
- Discharge to a long-term facility
- Involvement in a structured rehabilitation program
- Inability to perform lower or upper-body exercises (eg, being in wheelchair)
- Severe cardiac disease, including New York Health Association class III or IV congestive heart failure, clinically significant aortic stenosis, history of cardiac arrest, use of a cardiac defibrillator, or uncontrolled angina
- Diagnosed significant cognitive impairment, including known dementia diagnosis
- Organ transplant recipient on immunosuppressive agents
- HIV or AIDS with CD4+ T-lymphocytes count <200
- Severe immunocompromised state (eg, participant has neutropenia receiving cytotoxic chemotherapy with absolute neutrophil count <500/ul or expected to decline to <500/uL within the next 3 days)
- Progressive, degenerative neurologic disease (eg, Parkinson disease), multiple sclerosis
- Severe pulmonary disease, requiring either steroid pills or injections or the use of supplemental oxygen
- Simultaneous participation in another intervention trial
- No internet access in primary place of living

### Recruitment

This study is ongoing. The study suggested timeline is from May 1, 2023, to April 30, 2025. Table 1 provides a breakdown of data to be collected at each visit.

**Table 1:** Data collection summary by study visit.

Study phase	Hospital or home^a^	Hospital or home^a^	Home-based assessments		
Time-point description	Screening visit^a^ (V1)	Check-in visit^a^ (V2)	Baseline visit^a^ (V3)	Follow-up visit^a^ (V4)	12-week follow-up visit (V5)
Week	–8 to 0 weeks	–2 to 0 weeks	0 week	1 to 12 weeks	12 weeks (V5: –3 to + 7 days)
Method of the visits	In-person	In-person	Zoom call	Weekly call	Zoom call
Consent, review of inclusion or exclusion criteria	✓^a^				
Demographic and contact information	✓^a^				
Medical history review	✓^a^				
Medication inventory	✓ ^a^		✓		✓
Release of medical records authorizations form	✓^a^				
Depression questionnaire using CES-D^b^	✓^a^				
Short physical performance battery test	✓^a^				
Pregnancy test		✓		✓^c^	
Randomization		✓			
Distribute TheraBand		✓			
Distribute or collect tablets and pre-addressed return package		✓			✓
Update of medical history			✓		✓
MOCA/BLIND^d^			✓		✓
Quality of life questionnaire—EQ-5D-5L version (via app)			✓		✓
Timed up and go (via app)			✓		✓
30 second sit to stand test (via app)					
4 stage balance test (via app)					
Collection of adverse experiences	✓ ^a^		✓	✓	✓
ECOG^e^/WHO^f^/Zubrod Scale			✓		✓

^a^Screening visit (V1) and check-in visit (V2) may be scheduled on the same day for those patients recruited after hospital discharge.

^b^CES-D: Center for Epidemiologic Studies Depression Scale.

^c^We will send urine pregnancy tests every month for participants to complete at home. Pregnancy status will also be reassessed during the weekly calls.

^d^MOCA/Blind: Montreal Cognitive Assessment.

^e^ECOG: Eastern Cooperative Oncology Group.

^f^WHO: World Health Organization.

#### Participant Prescreening in the Electronic Health Records

Participants will be recruited at the University of Florida Health Shands Hospital in Gainesville, Florida, United States. Recruitment will start by reviewing admitted patient lists in the EPIC electronic health record (EHR) system (Epic Systems Corp). Participants must meet study criteria as well as qualify as septic using sepsis 3 criteria (sequential organ failure assessment score ≥2 compared to baseline with identified source of infection) [8]. All hospitalized patients with sepsis will be managed via the evidence-based management protocol that emphasizes early antibiotic administration, fluid resuscitation, and hemodynamic monitoring and support, consistent with current Surviving Sepsis Campaign guidelines [9,10]. Patients who screen positive for sepsis and are being treated by the standard-of-care sepsis protocol will be candidates for this study. Prescreening enables more patients to undergo screening since staff clinicians are unable to screen potential participants as part of their typical work duty.

In addition to the EPIC EHR review, clinical staff members, including physicians who serve as co-investigators or nurse coordinators, might recognize potential patients under their care and request the study team to assess their eligibility more thoroughly.

#### Confirmation of Eligibility

Once the clinical prescreening is complete, the study team physicians (also employed at the University of Florida Health Shands Hospital) or study staff (if a nonphysician clinical staff identified the potential participant) will confirm eligibility via a chart review.

#### Approaching Participants in the Hospital

Study staff will inform the clinical staff as to the potential participant’s eligibility and will request the clinical staff to approach the patient to gauge the patient’s interest in participating in the study. Once the patient expresses interest, the clinical staff will inform the study staff who will introduce the study to the patient more formally. If the study staff cannot speak with the patient directly to assess their study interest, either because of the patient’s location or time constraints, the study staff will ask a member of the patient’s clinical team, nurse or physician, to inquire about the patient’s interest in participating in the study. Patients will not be approached by the research coordinator until a member of their clinical staff has made initial contact and confirmation of their interest has been received by a member of the study team. If potential participants would like to learn more about the study but do not want to be approached by the study staff, they will be offered the study flyer that contains more information about the study and the study coordinators’ contact information and will be asked to contact the study coordinator if interested.

Research coordinators will approach the patients within 7 days before discharge or up to 3 days after discharge, for those patients who were discharged after business hours. The coordinator will consent the appropriate candidates and then further confirm their eligibility. Consent will be sought by coordinators, all of whom are familiar with institutional logistics and infrastructure and are experienced in the nuances of enrollment and informed consent for this challenging patient population. Coordinators will also monitor the clinical trajectory daily in the EPIC EHR and look for notes related to patient discharge planning. Consecutive patients meeting the criteria will be enrolled daily between 7 AM and 10 PM.

### Visit 1

Following the participants’ consent, they will undergo additional assessments to establish their eligibility, which will take place during visit 1 (also referred to as V1). Participants’ medical history and medication inventory will be reviewed for safety and to further confirm eligibility. Participants will also be asked to complete the SPPB which is an in-person physical function test comprising a 4-meter walk, repeated chair stands, and 3 increasingly difficult standing balance tests [11,12]. Participants scoring >10 points on the SPPB will be excluded from the study. Participants will also be screened for depression using the Center for Epidemiologic Studies Depression Scale (CES-D) questionnaire. If participants score≥16 on the CES-D, their clinical care team will be notified to provide them with appropriate care [13]. These participants will also be encouraged to discuss the results with their primary care physician. We will also collect demographic information as well as have participants sign a medical records release form. If the participant does not wish to schedule visit 2 (V2) at the end of visit 1, study staff will schedule a time to contact the participant per their request. Coordinators will leave their contact information with the participant.

#### Demographics, Medical History, and Medication Inventory

Participants will be asked to provide demographic information including birth date, address, phone number, sex at birth, gender, and ethnic background. Coordinators will use a case report form approved by the institutional review board to collect this information. Medical history will be assessed during prescreening but will be confirmed with the participant at V1 using a questionnaire. Participants will be asked a series of questions about their medical history to which they can answer “yes,” “no,” “I don’t know,” or “decline to answer.” Medication inventory will also be collected. Initially, outpatient medications will be assessed using the patient’s medical records, confirmed during V1, and will be confirmed with the patient.

### Visit 2

A second in-person home or hospital visit, V2, will be scheduled with each participant to explain all aspects of the study.

Participants will be randomized to either an intervention or a standard care control group immediately prior to this visit. All participants will be provided with tablets preloaded with both the Health in Motion and the Zoom apps. Before the visit, the study staff will ensure that all study devices are functional. The tablet will be Wi-Fi enabled for connection with a home internet network. Participants will receive in-person training and paper instructions on how to use and navigate Zoom call, tablet, and the Health in Motion app. The Zoom app will be used to connect with research study staff during the remote assessment visits. The participant will demonstrate understanding by reviewing the explained process with the study staff. Female participants who are aged 55 to 62 years with undetermined childbearing status per their medical records will be required to complete a urine pregnancy test during V2. Additionally, study coordinators will use information gleaned from conversations with the participant and their family members as well as a review of medical records to assess the participant’s ability to complete activities of daily living (ADL). In addition, we will use the Eastern Cooperative Oncology Group score, also called the Zubrod score (ECOG/Zubrod), to subjectively score their baseline abilities to complete ADL prior to hospitalization. Participants will also be given a prepaid or preaddressed box with packing supplies to mail any relevant study material back to the study team. In addition, participants randomized to the intervention group will receive rubber resistance bands (THERABAND) to add resistance to exercises as they progress.

### Remote Assessment Visits

Remote assessments will take place after the home visit and after –3 to +7 days of completion of the 12-week intervention phase. These visits are identified as baseline or V3 and 12-week follow-up or V5, respectively. Assessments will be conducted over Zoom by blinded study staff. Study staff will ensure the participant is performing the assessments properly and will visually monitor the safety of the participant. Assessments include a combination of physical function tests (TUG, 30STST, and 4SBT) and self-reported questionnaires (EQ-5D-5L, The Montreal Cognitive Assessment [MOCA-Blind], and ECOG/Zubrod).

#### Physical Function Assessments

Physical assessments include TUG [14], 30SSTS [15,16], and 4SBT [17] which are the primary efficacy outcomes for this study. These assessments are administered remotely through the Health in Motion app. The physical function tests will be completed at V3 and V5.

The TUG test [14] measures the time taken to stand from a seated position, walk 3 meters (about 10 feet), turn around, walk back, and sit down. This test measures fall risk, mobility, frailty, and lower extremity function. Two trials are completed and the faster of the 2 trials is the final score.

The 30SSTS [15,16] measures the number of times the participant can stand up from a seated position in 30 seconds. This test measures endurance and leg strength. The score is the total completed number of stands.

The 4SBT [17] measures an individual’s fall risk via their ability to hold static balance positions. This test consists of 4 stages of static balance positions, each progressively more difficult to maintain. Stage 1 is standing with feet touching side to side, stage 2 positions the feet in a semitandem position, stage 3 is full tandem, and stage 4 requires the individual to balance on 1 leg. In order to progress to the next stage, the static balance position must be maintained for 10 seconds without moving their feet or needing support. The total number of seconds a participant is able to hold the position for each stage becomes their score for a max score of 40. If a participant is not able to complete a stage, the test stops at this point and all subsequent stages are scored 0.

#### Self-Reported Questionnaires

At the initial screening visit (visit 3 [V3]) and the 12-week follow-up (visit 5 [V5]), a blinded study coordinator will administer questionnaires to evaluate participants’ cognitive function (MoCA/BLIND), quality of life (EQ-5D-5L), and self-perceived physical limitations (ECOG/Zubrod).

CES-D is a 20-item assessment tool that evaluates an individual’s depression symptoms by using commonly used self-statements that mirror their mental state. Participants will report the frequency with which they identify with these self-statements, such as “I felt hopeful about the future,” over the past week. Ratings are assigned on a 4-point Likert scale, ranging from 0=rarely or none of the time (0-1 days) to 3=most or all the time (5-7 days).

The MoCA/BLIND questionnaire serves as a screening tool for identifying cognitive impairment by evaluating various domains, including short-term memory recall, visuospatial abilities, executive functions, attention, concentration, working memory, language, and orientation to time and place. The MoCA-BLIND is an abbreviated telephone version that has no visual elements, scored out of 12 [18].

The EQ-5D-5L assessment will be administered using the Health in Motion app. It consists of inquiries about mobility, self-care, pain, usual activities, and psychological well-being, each with 5 response options (1=no problem, 2=slight problems, 3=moderate problems, 4=severe problems, and 5=unable). A summary index, with a maximum score of 1 will be generated from these 5 items using the US conversion table. The maximum score of 1 represents an optimal health state. Higher scores on individual indices reflect greater frequency and severity of issues.

#### Scored Questionnaires

Unblinded coordinators will use the ECOG/Zubrod scale during V2, V3, and V5 to characterize the patient’s level of functioning in terms of performing ADLs and physical ability. The ECOG/Zubrod scale consists of 5 categories numbered 0-5 with 0 being fully independent with no limitations, 4 being completely disabled (bed-bound, wheelchair-bound) and unable to perform self-care, and 5 as dead. The score is established by a study coordinator in the most unbiased manner through interviews conducted with both patients and their family members as well as chart reviews.

### Exercise Intervention

After the baseline visit, participants in the intervention group will be asked to begin the personalized exercise training program. This will consist of 12 weeks (5 days per week) of a guided exercise intervention program. Each session will consist of a 5-minute warm-up, followed by a 20-minute combination of aerobic and resistance or bodyweight exercises, stretching, and balance activities, and a 5-minute cooldown. In the early weeks of intervention, participants are mainly assigned 20-30 balance, stretching, and mobility activities with about one-third of exercises consisting of light strengthening activities including side-stepping, body weight rowing, and arm presses. There is equal emphasis on upper and lower body exercises and movement. As the intervention progresses throughout the 12 weeks, up to approximately 40 total exercises are assigned, with the goal of about half being more advanced strengthening exercises such as heel raises, chair push-ups, chest presses, and bicep curls using THERABANDs. Aerobic exercise mainly consists of walking, with progressively longer intervals assigned throughout the intervention.

According to the American College of Sports Medicine/American Heart Association guidelines, the intensity of exercise will be monitored using 10-point category-ratio scale, similar to the Borg scale, in which a score of 0=no physical exertion at all and 10=the extreme intensity of activity) [19,20]. Participants will be instructed to exercise at a self-perceived exertion level of approximately 3-4 and will be prompted to report their exertion level in the app throughout their daily exercise sessions. Coordinators will monitor their perceived exertion level and will adjust the intensity depending on participants’ progress and self-exertion score. The intensity of the resistance exercises will be increased with either the addition of the THERABANDs, an increase in the number of repetitions, and the elimination or reduction of a support mechanism for balance purposes.

#### Reminder Calls, Compliance, and Adherence

For reminder calls, all participants will receive regular phone calls to check on their health status, reminding them to charge the tablets, and to capture reports of adverse events. For the intervention group, participants will be contacted daily (5 days/week) for the first 2 weeks of the intervention. After 2 weeks for the intervention group and for the entire duration of the study for the control group, participants will be contacted weekly. We will reassess their childbearing status during the weekly calls, if applicable. If a participant reports that they could potentially be pregnant, they will be sent the urine pregnancy test via mail promptly to confirm their pregnancy status.

Compliance, for this study, is defined as the number of days exercising for 30 minutes per week; exercising 5 times/week for at least 30 minutes reflecting a 100% compliance.

Adherence, for this study, is defined as the number of days in which exercise was attempted or completed; logging into the app 5 times/week reflecting 100% adherence regardless of exercise time.

Compliance and adherence to their training schedule will be emphasized to the intervention group and they will be evaluated for any barriers if they miss 2 consecutive days or more of the exercise. Compliance and adherence will be monitored throughout the study duration remotely using the app’s administrative dashboard. Participants may experience complications that may impact compliance and adherence to the exercise program. Common causes of poor compliance and adherence include vacations, spousal care, fatigue, fluctuations in motivation, perceived lack of benefit, physical discomfort, and adverse health experiences.

#### Health Diary Entries

Participants in the control group will document their daily activities using the health diary feature available in the Health in Motion app. If any participant feels unsafe performing a test or their daily intervention program (if in the intervention group), they may skip the activity and input their reason within the textbox feature in the in-app diary.

### Safety

Several procedures will be adopted to ensure participant safety. Trained study personnel will explain potential adverse events and risks related to the study activities will be explained to the participants during the informed consent process by our trained study personnel. The study’s purpose and methods will also be explained in simple, easy-to-understand language. They will be encouraged to read the informed consent and ask questions. Adverse events will be collected and monitored by research staff during weekly calls and scheduled assessments. Participants will be encouraged to report the occurrence of adverse events in their in-app health diary within 48 hours of the occurrence. Participants will have access to study personnel 24 hours per day, 7 days a week to report serious adverse events or concerns regarding safety. They will be given the phone number of both the principal investigator and the lead research coordinator for urgent matters. Coordinators will also record adverse events that occur during functional assessments. The principal investigator and the study physician will review the reported adverse events to ensure the participants are safe to continue participating in the study. In consultation with the study physicians, a participant may be asked to seek medical attention or to schedule an in-person or telemedicine visit with the study physician.

Participants in the intervention group (group 1) will take part in warm-up and cooldown exercises to minimize discomfort and injury. Exercises will begin with low intensity and will gradually increase depending on the participant’s progress and reported exertion level in prior sessions. During V2 (in-home or hospital), participants will be advised to move slowly when attempting to rise from a seated or lying position as they are at risk of experiencing orthostatic hypotension. Further, participants will be instructed to stop the guided exercise when they feel pain, tightness, or pressure in the chest, significant shortness of breath, feeling faint, lightheaded, or dizzy, or significant other symptoms or medical problems. They will be instructed to report the event into the Health in Motion app and report it to the coordinator either immediately or in the next weekly call. The number of safety and adverse events will be tabulated as a primary safety outcome. At the end of the study, we will assess the safety of the proposed interventions by assaying the number of adverse events and serious adverse events reported in the exercise versus the control group.

Female participants aged 55 to 62 years will also complete urine pregnancy tests at home every month during the intervention period.

### Statistical Analyses

For qualitative measures, we will assess if the intervention is feasible, meaning relevant to this population and sustainable, by assessing participant feedback regarding the practicality of using this program, time commitment expectations, and ease of using the app among a diverse population in terms of age, sex, race, socioeconomic status, and baseline mobility. Safety will be evaluated by keeping in close contact with participants and asking them to report any discomfort, injury, or safety concerns.

The primary efficacy analysis will follow an “intent-to-treat” model [21]. For quantitative measures, differences in mean outcome measures and effect sizes between intervention groups will be calculated. Baseline characteristics of participants who do and do not have follow-up measures will be compared for missing data analysis. The relatively small sample size may imbalance prerandomization covariates [22]. Thus, caution will be taken in the interpretation of hypothesis tests.

### Ethical Considerations

#### Human Subject Ethical Review Approvals or Exemptions

Per institutional guidelines, this study has been reviewed and approved by the University of Florida’s institutional review board, under the project number IRB202200027. This study will operate under a partial Health Insurance Portability and Accountability Act waiver, allowing limited access to participant medical records only as deemed necessary by appropriately trained study staff. Study staff will minimize access to protected health information to what is absolutely necessary to determine candidacy for the study.

#### Informed Consent

During the informed consent process, participants are informed of their right to opt out of the study at any time with no penalties. For secondary analyses using existing data, we do not require additional consent. Study data are deidentified at the analysis phase, for additional scenarios where informed consent may not be required.

#### Privacy and Confidentiality

Each participant is given a unique identifier (HS0X with X being enrollment order in ascending order). The participant’s name is collected and kept in their binder, but data when input to REDCap (Vanderbilt University) are deidentified. Binders are kept under double-lock conditions. After the closure of the study, identifying information will be removed from the participant’s binder.

#### Compensation Details

Participant payment outlines are discussed in the informed consent process including all potential payments they can be eligible for should they complete the study, payment schedule, and method of payment. Participants are paid US $50 during their baseline Zoom visit, US $50 at 4 weeks, US $50 at 8 weeks, and US $150 once they have completed the 12-week follow-up visit and returned the tablet via mail. If a participant has to travel to complete a screening visit within 72 hours of enrollment, they will be compensated an additional US $10. There are no other exceptions for the rate of payment, as long as a participant continues to comply with study guidelines they are compensated as stated.

## Results

Previous research studies suggest that physical activity interventions prevent mobility disability in rather healthy older adults [1,18,23]. However, older sepsis survivors are frailer, more sedentary, and lower-functioning, and are more likely to struggle to travel to exercise or rehabilitation facilities to receive supervised exercise training programs than older adults of the same age [7,24]. To our knowledge, this will be the first home-based therapeutic approach with remote therapeutic monitoring, to improve the physical function of older sepsis survivors using a 12-week exercise program delivered via a digital health platform. The goal of this study is to evaluate the safety and feasibility of this remotely delivered exercise program. Additionally, it will assess the efficacy of the intervention and provide descriptive estimates of effects, and nominal estimation of the mean changes in physical function. Evaluating feasibility through this study will also help to the assess ease of recruitment and retainment of participants, adherence to study intervention protocol, and safety by assessing the percentage of serious adverse events reported. These data will be used to estimate the power needed for a larger randomized clinical trial. As of August 2024, 15 eligible patients have been enrolled in the study. Screening and recruitment are still ongoing and are estimated to be completed by January 2025.

## Discussion

The study design is built upon the work of similar studies but with a few novel features. Several studies have shown that mainly home-based exercise programs delivered over several weeks with progressively more difficult exercises are efficacious at improving physical function in older adults with [3,25,26] and without chronic illness [18,27]. Many of these studies are home based but are not remotely or digitally delivered like our study. We anticipate that this will enhance the program’s accessibility for individuals facing geographical barriers or transportation challenges, residing in remote regions, or those for whom physical intervention programs have not been recommended by their health care providers.

Furthermore, drawing from comparable research on home-based exercise regimens for older adults and individuals recovering from critical illness, our exercise program advances participants through increasingly challenging exercises. However, we have added a novel component with the ability to adjust exercise difficulty based on perceived exertion rating scales. In fact, this was identified as a limitation from a study closely resembling ours [2].

The participants are another unique aspect of our study. We are unaware of any home-based exercise program studies whose population solely consists of patients hospitalized for sepsis. This builds upon existing literature which has shown that in-hospital rehabilitation is associated with less physical disability once released from the hospital [20,28]. Often, these patients do not continue the momentum of exercise at home. It must be noted that there is also a need for sepsis-specific studies, as compared to patients hospitalized for other reasons because those hospitalized for sepsis have higher rates of physical disability when discharged [29-31].

Another notable limitation of previous studies is the lack of baseline data, which made it difficult to identify individuals who would benefit the most from rehabilitation after critical illness [2,3]. This challenge is addressed in our study design. Baseline data are measured using several methods that have been validated by experts [4-6,24] for comparison at follow-up evaluation. We will also prevent patients who may be physically fit and will not receive extra benefits from this study by excluding those who score less than 10 on the SPPB at the screening encounter.

In addition to many novel features, there are some notable strengths to our study design. To mitigate anticipated issues with adherence, our team will be in regular contact with participants and will ask specific questions to facilitate identifying and intervening on issues before they arise. Additionally, our team will use a feature on the Blue Marble Health app that allows for monitoring of compliance with daily exercise. A social problem-solving approach will be used to identify and address compliance issues. To mitigate potential dropout, all study procedures will be conducted remotely or are designed to be convenient for participants. V2 will occur while the patient is in the hospital, or the study staff will travel to the participants’ homes to provide them with study materials and instructions for use. Otherwise, the entire program including assessments and check-ins is delivered remotely. Additionally, our team meets weekly to discuss concerns and strategies to proactively address problems.

As for the study limitations, first, enrollment of participants in the critical care hospital setting will be challenging. This population is often too ill to participate in an exercise intervention program, and many are referred to outpatient rehabilitation or nursing facilities where they will receive PT or OT. Another pertinent limitation is the lack of true baseline data regarding the prior level of function. Study staff will review participants’ medical records and collateral may be obtained from friends and family but ultimately all information is subjective. Finally, although we will be able to monitor how much time is spent on each exercise and how many repetitions are completed, there is no definitive way to confirm if the exercises are being performed correctly or at the intensity required to improve physical function, which presents a limitation for monitoring the participants’ true exercise participation.

Collectively, the concepts, methodology, and interventions involved in this study are novel to the field of critical care medicine, geriatrics, and rehabilitation. As sepsis is often associated with high rates of morbidity and mortality, understanding how to minimize these effects through rehabilitation is critical to older individuals’ long-term health outcomes and quality of life. If our program is found to be safe, efficacious, and feasible for use in older sepsis survivors, we hope to further assess viability of this program in improving physical function outcomes using a larger sample size.

## Appendix

Multimedia Appendix 1 Peer review report from the Aging Systems and Geriatrics (ASG) Study Section - Brain Disorders and Clinical Neuroscience Integrated Review Group - Center for Scientific Review (National Institutes of Health, USA).

## Abbreviations

30SSTS: 30 second sit to stand test
4SBT: 4 stage balance test
ADL: activities of daily living
CES-D: Center for Epidemiologic Studies Depression Scale
ECOG: Eastern Cooperative Oncology Group
EHR: electronic health record
MOCA/Blind: Montreal Cognitive Assessment
OT: occupational therapy
PT: physical therapy
SPPB: short physical performance battery
TUG: timed up and go test
V1: visit 1
V2: visit 2
V3: visit 3
V5: visit 5
