# Supplementary material for: Home-Based Digital Exercise Training Program to Improve Physical Function of Older Sepsis Survivors: Protocol of the HEAL Sepsis Randomized Clinical Trial
Source: JMIR Res Protoc. 2024 Oct 17;13:e60270. doi: 10.2196/60270 (PMC11528160; doi:10.2196/60270)
Supplement: Multimedia Appendix 1 [file resprot_v13i1e60270_app1.pdf]

1R21AG075645-01 MANKOWSKI, ROBERT

RESUME AND SUMMARY OF DISCUSSION: In this R21 application, the applicant proposes to test the safety, feasibility and efficacy of a novel 12-week physical activity intervention to improve physical function in older sepsis survivors utilizing an application-based digital exercise training platform in randomized 40 sepsis survivors (aged >65 years) with an SPPB score <6 at the time of their discharge to home from the hospital into the intervention or a standard care control group that will receive a tablet with the Blue Marble Health Platform (BMHP) application which contains all of the physical function assessments, and a health diary including personalized avatar-guided exercises as well as text message reminders to exercise for 30min each day, 5 days/week for the study group. If successful, the results could provide information regarding the feasibility, tolerability, and efficacy of this novel approach to enhance physical function in older sepsis survivors. During the discussion, the reviewers noted that the research question was important and significant. The foundational science was rigorous derived from a recognized gap from the literature and observations from their database. There was novelty in the approach and the experimental design, methodology and statistical evaluation plan were good. Though the PI was relatively junior, he was experienced and well funded, and supported by appropriate senior colleagues within an excellent research environment. There was some concern that the team lacked prior experience with avatar training, and there were insufficient details regarding the training and participant familiarity and access to reliable internet, and validity of self evaluation and reporting. Nevertheless, there was considerable enthusiasm for this excellent application, the results from which could potentially have high impact on the application of home exercise programs for older sepsis survivors.

DESCRIPTION (provided by applicant): Sepsis, an exaggerated response to infection, affects all ages, but it is more prevalent in older adults. Older sepsis survivors are commonly discharged to long-term acute care facilities, where they often die within 1 year. We believe that those who return home from the hospital lose the momentum of physical function improvement after early in-hospital rehabilitation and often face exacerbation of comorbidities and decline in physical function. Based on our longitudinal observational data, older sepsis survivors have poor health status and physical function lasting at least 1 year after sepsis, and in contrast with younger survivors, older patients do not regain their function. Our aging research suggests that physical activity interventions prevent mobility disability in rather healthy older adults, but older sepsis survivors will be profoundly frailer, more sedentary, lower- functioning and will unlikely be capable of traveling to participate in supervised exercise training programs in our research facilities. Currently, we are unaware of any home-based therapeutic approaches to improve physical function of older sepsis survivors. Current mobile health applications can deliver structured home-based exercise programs to reach daily activity goals. For example, the Blue Marble Health Platform (BMHP), developed and validated with the support of NIH contains over 100 avatar-guided strengthening, balance, and aerobic exercises, along with 23 standardized assessments and a personal health diary. This digital and remotely monitored exercise training program might be promising for sepsis survivors with poor health status, who are unlikely to be able to travel to participate in exercise interventions. Our proposed study will test the safety, feasibility and efficacy of a novel 12-week physical activity intervention to improve physical function in older sepsis survivors utilizing an app-based digital exercise training platform. We propose to randomize 40 sepsis survivors (aged >65 years) with an SPPB score <6 at the time of their discharge to home from the hospital into the intervention or a standard care control group. Both groups will receive a tablet with the BMHP app, which contains all of the physical function assessments, and a health diary. The intervention group will also receive personalized avatar-guided exercises as well as text message reminders to exercise for 30min each day, 5 days/week. For the control group, participants will record

their daily activities using the BMHP health diary. A research coordinator will call all participants weekly to ask questions about their health status and remind them to charge the tablets. At baseline and 12-week follow-up, participants will use the app to complete the app- guided physical function assessments and we will measure activity levels using hip-worn accelerometers. This project will be the first to test the safety, feasibility and efficacy of a digital physical activity intervention in this high-risk population of older sepsis survivors. The results will provide critical information regarding the feasibility, tolerability, and efficacy of this novel approach to enhance these patients' physical function.

PUBLIC HEALTH RELEVANCE: Older adults are affected by sepsis more often than younger patients and, even more worrisome, have a higher risk of physical disability and death within 1 year after sepsis. While these older sepsis survivors need therapeutic approaches to improve their physical function after returning to home to continue their momentum of physical function improvement initiated by early physical/occupational therapy in hospital, supervised exercise training programs might not be feasible due to the patients' poor health status and limited access to physical therapy/fitness facilities. To address this issue, our proposed study will test a novel and practical home-based, digitally delivered physical activity intervention that does not require visits to intervention sites and is tailored to each participant's functional status and progress.

CRITIQUE 1

Significance: 1
Investigator(s): 1
Innovation: 1
Approach: 1
Environment: 1

Overall Impact: This R21 application proposes to study a home-based digital exercise training program to improve physical function of older sepsis survivors dubbed as "HEAL Sepsis Trial". It proposes to test the central hypothesis that a 12-week digital, home-based, remotely-monitored intervention that guides physical activity will be feasible and efficacious in improving physical function in older sepsis survivors with two specific aims – aim 1 tests feasibility and 2 tests efficacy. The study proposes to address a critically important clinical issue for post-acute rehabilitation of older patients after surviving from sepsis. While it is high risk, the high yield relies on its potential for paradigm shift on this very much needed, but unexplored clinical and research area. Therefore, it's highly significant and innovative. The innovation and feasibility are also demonstrated by taking the advantage of internet-based technology and telemedicine whose development has been accelerated after COVID-19 pandemic. The PI is an Assistant Professor with excellent funding and publication track record. The research team also includes senior faculty. The PI has extensive experience in such trials and the approach for the proposed study is solid. Since sepsis is a very diverse clinical entity, adding expertise in infectious diseases and perhaps also critical care would further strengthen the application. In addition, it's unclear whether 12-week intervention would be enough duration and effective ways to follow-up after the intervention so that functional gains can be maintained.

1. Significance:

Strengths

- Critically important clinical issue for post-acute rehabilitation of older patients after surviving from sepsis.

- While it is high risk, the high yield relies on its potential for paradigm shift for post-acute rehabilitation for this older patient population.

Weaknesses

- None noted.

2. Investigator(s):

Strengths

- The PI is an Assistant Professor with extensive training and experience in the proposed research area and similar trials.
- The PI has excellent funding and publication track record.
- The research team also includes senior faculty and biostatistics.

Weaknesses

- Given that sepsis is a very diverse clinical entity, adding expertise in infectious diseases and perhaps also critical care would further strengthen the application.

3. Innovation:

Strengths

- Home-based physical exercise program for this particular patient population is rare in the current post-acute rehabilitation paradigm. Therefore, such study is novel.
- The study is also innovative as it takes the advantage of internet-based technology and telemedicine whose development has been accelerated after COVID-19 pandemic.

Weaknesses

- None noted.

4. Approach:

Strengths

- Solid research design and approach with detailed description of recruitment, inclusion and exclusion criteria, randomization, and study protocol.
- Description of power calculation and analytical plan as well as potential pitfalls and alternative ways to address them.

Weaknesses

- Providing details justifying why 12-week intervention would be enough and description of follow-up strategy after the intervention so that functional gains can be maintained would further strengthen the application.

5. Environment:

Strengths

- Outstanding research environment at UF, particularly with exercise physiology.

Weaknesses

- None noted.

Study Timeline:
Strengths
- Recruitment and study timeline are appropriate for the proposed R21.

Weaknesses
- None noted.

Protections for Human Subjects:
Acceptable Risks and/or Adequate Protections
- Addressed

Data and Safety Monitoring Plan (Applicable for Clinical Trials Only):
Acceptable
- Included.

Inclusion Plans:
- Sex/Gender: Distribution justified scientifically
- Race/Ethnicity: Distribution justified scientifically
- For NIH-Defined Phase III trials, Plans for valid design and analysis: Not applicable
- Inclusion/Exclusion Based on Age: Distribution justified scientifically
- The study has a scientific focus on older sepsis patents.

Vertebrate Animals:
Not Applicable (No Vertebrate Animals)

Biohazards:
Acceptable
- Addressed.

Applications from Foreign Organizations:
Not Applicable (No Foreign Organizations)

Select Agents:
Acceptable

Resource Sharing Plans:
Acceptable

Authentication of Key Biological and/or Chemical Resources:
Acceptable

Budget and Period of Support:
Recommend as Requested

CRITIQUE 2

Significance: 2
Investigator(s): 2
Innovation: 3
Approach: 4
Environment: 2

Overall Impact: This R 21 proposes to use a 12 week digital home based remotely monitored intervention to guide sepsis survivors through physical activity exercises. Strengths of the application include a focus on a vulnerable subpopulation of sepsis survivors- those that cannot participate in intensive outpatient physical or occupational therapy.

1. Significance:

Strengths

- Home based care of older adult post- discharge is increasingly attractive from safety, cost, scalability perspectives.
- Home based care of older adults, if applied equitably, could reduce disparities in post-acute care and post sepsis recovery.

Weaknesses

2. Investigator(s):

Strengths

- The investigators are well suited for the conduct of this type of study.

Weaknesses

3. Innovation:

Strengths

- This proposal focuses on relatively unexplored area in post critical illness research-the portion of illness survivors who are too dysfunctional or disabled to benefit from traditional, guideline based care.

Weaknesses

- Mobile based health applications rely on a senior is ability to interact with technology, have social support allowing them to interact with technology. By default, this may result in the exclusion of some of the members of the vulnerable group this proposal seeks to target.

4. Approach:

Strengths

- The scope of the proposed research is limited to feasibility and safety.
- The preliminary data is excellent inappropriate to the research questions.
- Although limited, the review of previous literature is adequate.

Weaknesses

- Reliance on individual ability to manage tablets with applications will result in channeling of the intervention, potentially limiting the generalizability of the findings.

5. Environment:

Strengths

- The Sepsis and Critical illness Research Center has a large database consisting of longitudinal data collected on 320 sepsis survivors. This is a unique resource.

Weaknesses

Protections for Human Subjects:

Acceptable Risks and/or Adequate Protections

Data and Safety Monitoring Plan (Applicable for Clinical Trials Only):

Acceptable

Inclusion Plans:

- Sex/Gender: Distribution justified scientifically
- Race/Ethnicity: Distribution justified scientifically
- For NIH-Defined Phase III trials, Plans for valid design and analysis:
- Inclusion/Exclusion Based on Age: Distribution justified scientifically

Vertebrate Animals:

Not Applicable (No Vertebrate Animals)

Biohazards:

Not Applicable (No Biohazards)

Applications from Foreign Organizations:

Not Applicable (No Foreign Organizations)

Select Agents:

Not Applicable (No Select Agents)

Resource Sharing Plans:

Acceptable

Authentication of Key Biological and/or Chemical Resources:

Not Applicable (No Relevant Resources)

Budget and Period of Support:

Recommend as Requested

Recommended budget modifications or possible overlap identified:

- Requested direct cost were less than $250,000 per year.

CRITIQUE 3

Significance: 1
Investigator(s): 4
Innovation: 3
Approach: 4
Environment: 3

Overall Impact: This is a good proposal that will use Avatar based exercise programs for at home exercise in sepsis survivors. The significance of the study is that increasing physical activity in these patients has the ability to greatly improve health care outcomes in this vulnerable population. The panel of avatar exercises has already been developed and the study is designed to test the hypotheses. Enthusiasm for this proposal is limited by the lack of expertise in the study team with avatar use, especially in older adults. It is not clear if this patient population can/will use the application appropriately. The plan for training the older adults to use the electronic devices in this study is vague, and yet critical for the success of the study.

1. Significance:

Strengths

- Remote delivery of physical activity programs in vulnerable populations such as sepsis survivors is important.

Weaknesses

2. Investigator(s):

Strengths

- Mankowski (PI) has experience with aging populations including sepsis.

- Investigative team has clinical experience with sepsis and sepsis survivors.

Weaknesses

- No one on the team appears to have publications or experience with avatars and delivery of wellness programs using avatars.

3. Innovation:

Strengths

- The ‘hands off’ application that will provide the physical activity intervention.

Weaknesses

4. Approach:

Strengths

- The recruitment and characterization of the sepsis survivors is a strength of this study.
- The experimental and control group are well characterized and appropriate.
- The outcome measures are adequate, including adherence to the intervention, and differences in 7-day physical activity between the groups.

Weaknesses

- Given the inexperience of the investigation team with avatar health care delivery, it is not clear if the right exercises will be chosen, or that the intervention can be monitored and interpreted adequately.
- The initial training session(s) provided to the participants is not explained. How long will it take to prepare the participants in each group? Training is needed to use the devices and perform data recording, as well as the various exercises. Will training include where and when to exercise? How will success in training be documented?
- The study team will depend on core resources to analyze and interpret accelerometer data.

5. Environment:

Strengths

- The SCIRC can provide a target for recruitment.
- The IOA and CTRB can provide support for this project.

Weaknesses

- Connection to expertise and resources related to remote health care delivery or avatar use was not presented.

Study Timeline:

Strengths

- Clearly set up.

Weaknesses

- IRB approval scheduled to be completed in the first quarter of the study

Protections for Human Subjects:
Acceptable Risks and/or Adequate Protections
Data and Safety Monitoring Plan (Applicable for Clinical Trials Only):
Acceptable

Inclusion Plans:
- Sex/Gender: Distribution justified scientifically
- Race/Ethnicity:
- For NIH-Defined Phase III trials, Plans for valid design and analysis:
- Inclusion/Exclusion Based on Age: Distribution justified scientifically
- Will study older adults as they are felt to be most at risk for inactivity related complications.
- Expect equal numbers of men and women.
- Will recruit race/ethnicity based on patient population.

Vertebrate Animals:
Not Applicable (No Vertebrate Animals)

Biohazards:
Not Applicable (No Biohazards)

Applications from Foreign Organizations:
Not Applicable (No Foreign Organizations)

Select Agents:
Not Applicable (No Select Agents)

Authentication of Key Biological and/or Chemical Resources:
Not Applicable (No Relevant Resources)

Budget and Period of Support:
Recommend as Requested

THE FOLLOWING SECTIONS WERE PREPARED BY THE SCIENTIFIC REVIEW OFFICER TO SUMMARIZE THE OUTCOME OF DISCUSSIONS OF THE REVIEW COMMITTEE, OR REVIEWERS' WRITTEN CRITIQUES, ON THE FOLLOWING ISSUES:

PROTECTION OF HUMAN SUBJECTS: ACCEPTABLE

INCLUSION OF WOMEN PLAN: ACCEPTABLE

INCLUSION OF MINORITIES PLAN: ACCEPTABLE

INCLUSION ACROSS THE LIFESPAN: ACCEPTABLE

COMMITTEE BUDGET RECOMMENDATIONS: The budget was recommended as requested.

Footnotes for 1 R21 AG075645-01; PI Name: Mankowski, Robert T

+ Derived from the range of percentile values calculated for the study section that reviewed this application.

NIH has modified its policy regarding the receipt of resubmissions (amended applications).See Guide Notice NOT-OD-18-197 at https://grants.nih.gov/grants/guide/notice-files/NOT-OD-18-197.html. The impact/priority score is calculated after discussion of an application by averaging the overall scores (1-9) given by all voting reviewers on the committee and multiplying by 10. The criterion scores are submitted prior to the meeting by the individual reviewers assigned to an application, and are not discussed specifically at the review meeting or calculated into the overall impact score. Some applications also receive a percentile ranking. For details on the review process, see http://grants.nih.gov/grants/peer_review_process.htm#scoring.
